# *Oestrus ovis* L. (Diptera: Oestridae) Induced Nasal Myiasis in a Dog from Northern Italy

**DOI:** 10.1155/2016/5205416

**Published:** 2016-08-01

**Authors:** Sergio A. Zanzani, Luigi Cozzi, Emanuela Olivieri, Alessia L. Gazzonis, Maria Teresa Manfredi

**Affiliations:** ^1^Dipartimento di Medicina Veterinaria, Università degli Studi di Milano, Via Celoria 10, 20133 Milano, Italy; ^2^Dipartimento di Medicina Veterinaria, Università degli Studi di Perugia, Via S. Costanzo 4, 06126 Perugia, Italy

## Abstract

A companion dog from Milan province (northern Italy), presenting with frequent and violent sneezing, underwent rhinoscopy, laryngoscopy, and tracheoscopy procedures. During rhinoscopy, a dipteran larva was isolated from the dog and identified as first instar larval stage of* O. ovis* by morphological features. Reports of* O. ovis* in domestic carnivores are sporadic and nevertheless this infestion should be considered as a possible differential diagnosis of rhinitis in domestic carnivores living in contaminated areas by the fly as consequence of the presence of sheep and goats. This report described a case of autochthonous infestion in a dog from an area where* O. ovis* was not historically present but it could be affected by a possible expansion of the fly as a consequence of climate change. This is the first record of* Oestrus ovis* infestion in a dog in Italy and, at the same time, the most northerly finding of larvae of sheep bot fly in the country.

## 1. Introduction

*Oestrus ovis* L. (Diptera: Oestridae), a sheep nasal bot fly, affects sheep and goats worldwide and, particularly, in areas where adult flies can be active all the year round thanks to favourable climatic conditions [1]. In central and southern Italy prevalence of infestion in sheep is high: 55.8%, 72.8%, and 91% of infected sheep were observed during necropsies in Sicily [2], Tuscany [3], and Sardinia [4], respectively. Zoonotic infestions sustained by* O. ovis* are numerous and diffused all over the world. In Italy,* O. ovis* infestions in humans were first described in Sicily in the 19th century [5] and several infestions have been reported even in more recent years, mostly in southern rural areas [6]. Sporadic descriptions of zoonotic infestions by* O. ovis* are reported also in central [7] and northern Italy [8, 9] as well as in an urban area [10].* O. ovis* infestions in ovine and humans in the most northerly parts of Italy are reported below 45 degrees north latitude: in Liguria, Emilia-Romagna, and northern Tuscany. Reports of* O. ovis* infestions in domestic carnivores are sporadic [11–16] and have not been yet described in Italy. The aim of the present study is to describe an autochthonous case of* O. ovis* infestion in a companion dog bred in northern Italy.

## 2. Case Presentation

**Figure 1:**
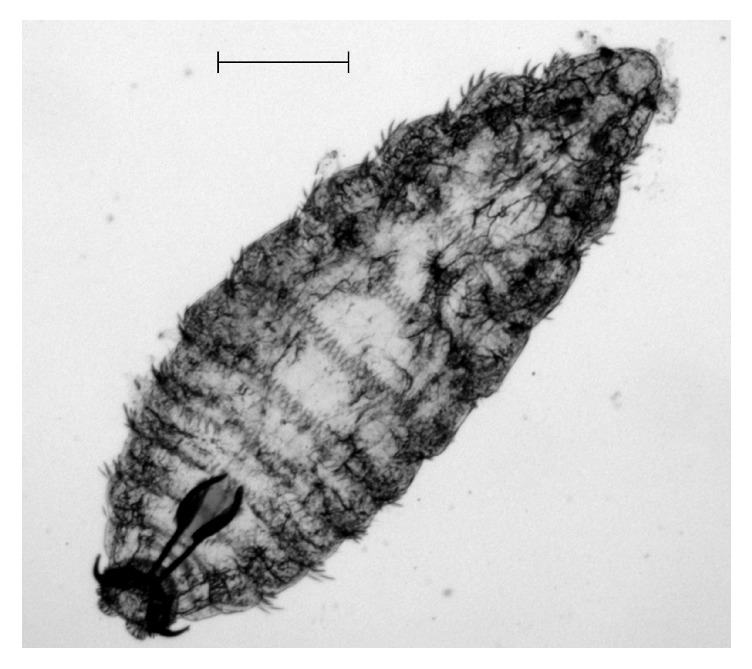
*O. ovis* L1 collected from the dog after nasal lavage (40x). Black bar indicates 200 *μ*m length.

**Figure 2:**
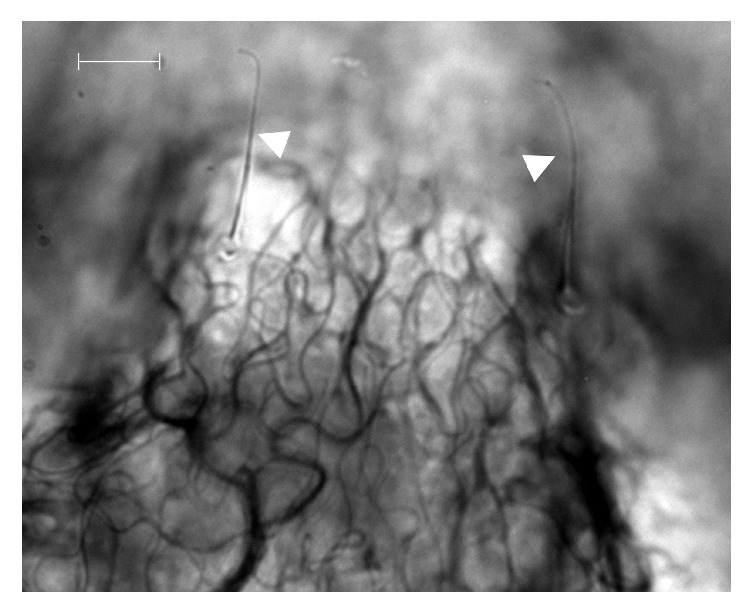
*O. ovis* L1 surface (630x). White arrows indicate cuticular sensilla and white bar indicates 10 *μ*m length.

**Figure 3:**
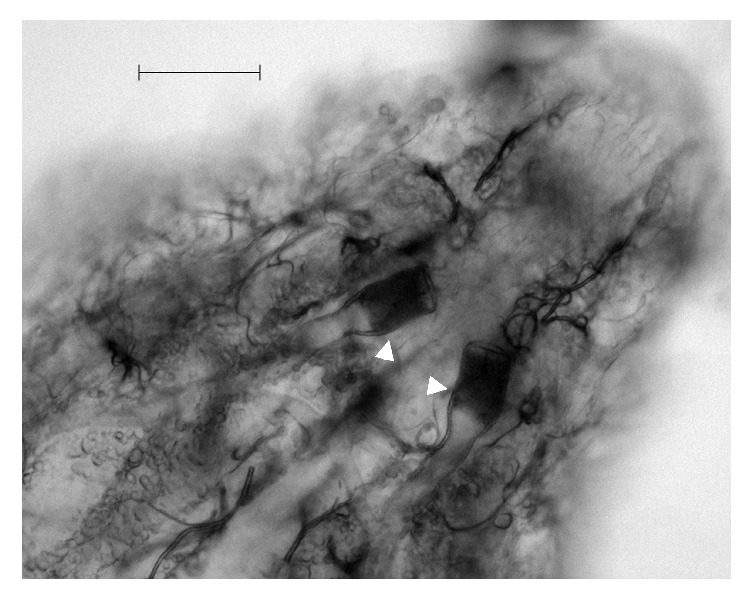
Terminal segments of* O. ovis* L1 (200x). White arrows indicate tracheal trunks and black bar indicates 50 *μ*m length.

In July 2015, an 8-month-old female of Staffordshire Bull Terrier, housed in Milan province (northern Italy) and purchased from an Italian dog breeder, was taken to a veterinary clinic on account of her frequent and violent sneezing that lasts for two days. During anamnestic data collection, the owner reported that sneezing occurred after the dog had been taken for a walk in a rural area close to his house. At clinical examination the bitch also presented stertorous and reversal sneezing. Anamnesis, dog breed, and symptoms made clinicians suspect a nasal foreign body and/or a brachycephalic airway obstructive syndrome (BAOS). No antimicrobial or anti-inflammatory therapies were being administered to the dog. The bitch was then anesthetized for laryngoscopy, tracheoscopy, and anterior and posterior rhinoscopy. Laryngeal inspection revealed everted laryngeal saccules, whereas tracheoscopy did not show any remarkable alteration. Posterior rhinoscopy evidenced few small mucosal erosions (diameter < 2 mm) surrounded by mildly thickened and oedematous mucosae in the rhinopharynx; a small quantity of mucus-like material was also present. The anterior rhinoscopy highlighted two and three whitish fusiform organisms in the right and in the left nasal cavities, respectively; all the observed organisms appeared to be vital, presenting high mobility on the nasal mucosal surface. Attempts to catch them using endoscopic forceps failed and only after nasal lavage was one of them isolated and collected. Noticeably, following nasal lavage, the acute and violent sneezing improved considerably which might be due to removal of most of the observed organisms. The collected organism resembled a larva of Diptera and while waiting for further investigations after rhinoscopy the dog was also treated for three times every 7 days (days 0, 7, and 14) with subcutaneous administration of 300 *μ*g/kg of ivermectin. After treatment, sneezing disappeared completely, and only moderate reversal sneezing, probably due to everted laryngeal saccules, remained present. The larva was sent to the Department of Veterinary Medicine of Milan for identification; it was studied under the light microscope and identified according to morphological keys [17–21]. The specimen was identified as a first instar larval stage (L1) of* O. ovis* L. (Diptera: Oestridae). The fusiform and dorsoventrally flattened L1, about 1.18 mm long and 0.44 mm wide, was divided into 11 segments (Figure 1). On its surface, these segments presented trichoid cuticular sensilla (Figure 2). Such structures are thermosensitive; they allow L1 to both locate and, in association with its quick mobility, rapidly reach the nasal cavities to find a suitable niche for its development. Ventral and lateral clusters of spines were also evident on the larva surface. They measured about 20 *μ*m and 30 *μ*m in length, respectively, and their distribution resembled the typical pattern described in* Oestrus* larvae. In subfamily Oestrinae, lateral and ventral spines can help a larva attach to and move on the host's mucosal surface without being expelled by its sneezing. The larva under investigation showed a distinctive cluster of spines on the terminal abdominal segment, though its bilobated shape was not perfectly preserved. Cranially, a pair of prominent, dark brown oral hooks, connected to the internal cephalopharyngeal skeleton, as well as defined antennal lobes, measuring about 18 × 22 *μ*m could be noticed. Broad tracheal trunks, about 20 *μ*m wide, ended between the tenth and eleventh body segments (Figure 3).

## 3. Discussion

*O. ovis* is an agent of myiasis in sheep and goats. Members of Oestridae family tend to be highly host-specific, with preference for herbivores. We described the first record of* O. ovis* infestion in a dog in Italy and, at the same time, the most northerly finding of larvae of sheep bot fly in our country. The infected dog we examined lives in a village near Milan and might have come down with the infestion in an area located about 60 km north of the 45th parallel north. Reasonably, it is a case of autochthonous infestion. In fact, contacts between sheep parasites and companion dogs are likely to occur because even though Milan with its territory is highly urbanized rural areas crossed by transhumant flocks from the PreAlpine areas are still present. Furthermore, unlike other regions such as Tuscany, Liguria, and Emilia Romagna, Lombardy is not characterised by immigration of shepherds and flocks from Sardinia or southern Italy; thus, it can be hypothesised that the presence of sheep nasal bot fly in the studied area is a consequence of temperature increase as observed in several surveys conducted in northern Italy [22–24]. Climate change there might have favoured a habitat more suitable to adult flies survival, as emphasized by some authors [25]. This hypothesis is also supported by the findings (after the record of the infestion in the dog) of other cases of* O. ovis* infestion in small ruminants bred in three provinces (Bergamo, Varese, and Brescia) located to the north of Milan and referred to our laboratories.

As to dogs, it should be noted that in domestic carnivores (dogs and cats)* O. ovis* infestions are less common than in humans, having been sporadically described in dogs from India [11], Spain [12, 13], New Zealand [14], and UK [16] and in a cat from Australia [15]. Low occurrence of* O. ovis* infestions in domestic carnivores might be due to peculiar sheep bot fly preferences and, in general, due to the strong relationship between oestrids and herbivores. In fact, the only species belonging to Oestridae family that naturally infects carnivores is* Dermatobia hominis*, although it mainly parasitizes herbivores [19]. Moreover, in rural areas and in developing countries, infestions in dogs and cats could go unnoticed or undetected most likely because an* in vivo* diagnosis of nasal myiasis in carnivores is possible only if larvae and/or puparia are collected by pet owners or clinicians and correctly identified. It is a fact that no serological tests are available for dogs and cats and the described symptoms of infestion are nonspecific (i.e., sneezing, stertor, nasal discharge, excitation, loss of appetite, coughing fits, unilateral epistaxis, and fever). Then, collection of larvae and/or puparia* in vivo* can be performed only if spontaneous expulsion from nasal cavities through nostrils is noticed by pet owners or occurs during a diagnostic procedure such as a rhinoscopy.

Thus, in case of rhinitis in domestic carnivores, nasal myiasis due to* O. ovis* should be considered as a possible differential diagnosis, especially when proximity to small ruminant farms is reported in the anamnesis and usual antimicrobial and/or anti-inflammatory treatments result to be ineffective.
